# Conventional Chemotherapy and Oncogenic Pathway Targeting in Ovarian Carcinosarcoma Using a Patient-Derived Tumorgraft

**DOI:** 10.1371/journal.pone.0126867

**Published:** 2015-05-11

**Authors:** Gretchen Glaser, S. John Weroha, Marc A. Becker, Xiaonan Hou, Sergio Enderica-Gonzalez, Sean C. Harrington, Paul Haluska

**Affiliations:** 1 Division of Gynecologic Surgery, Mayo Clinic, Rochester, MN, United States of America; 2 Department of Oncology, Mayo Clinic, Rochester, MN, United States of America; University of South Alabama, UNITED STATES

## Abstract

Ovarian carcinosarcoma is a rare subtype of ovarian cancer with poor clinical outcomes. The low incidence of this disease makes accrual to large clinical trials challenging. However, studies have shown that treatment responses in patient-derived xenograft (PDX) models correlate with matched-patient responses in the clinic, supporting their use for preclinical testing of standard and novel therapies. An ovarian carcinosarcoma PDX is presented herein and showed resistance to carboplatin and paclitaxel (similar to the patient) but exhibited significant sensitivity to ifosfamide and paclitaxel. The PDX demonstrated overexpression of EGFR mRNA and gene amplification by array comparative genomic hybridization (log2 ratio 0.399). EGFR phosphorylation was also detected. Angiogensis and insulin-like growth factor pathways were also implicated by overexpression of VEGFC and IRS1. In order to improve response to chemotherapy, the PDX was treated with carboplatin/paclitaxel with or without a pan-HER and VEGF inhibitor (BMS-690514) but there was no tumor growth inhibition or improved animal survival, which may be explained by a *KRAS* mutation. Resistance was also observed when the IGF-1R inhibitor BMS-754807 was combined with carboplatin/paclitaxel. Because poly (ADP-ribose) polymerase inhibitors have activity in ovarian cancer patients, with and without *BRCA* mutations, ABT-888 was also tested but found to have no activity. Pathogenic mutations were also detected in *TP53* and *PIK3CA*. In conclusion, ifosfamide/paclitaxel was superior to carboplatin/paclitaxel in this ovarian carcinosarcoma PDX and gene overexpression or amplification alone was not sufficient to predict response to targeted therapy. Better predictive markers of response are needed.

## Introduction

Ovarian cancer (OC) is the most lethal gynecologic malignancy, partly due to the lack of reliable screening methods and advanced stage at initial diagnosis. Ovarian carcinosarcoma (OCS), one of the most rare and aggressive forms of OC, remains extremely difficult to treat [1]. In the absence of randomized data, chemotherapy options for patients with OCS are based primarily on data from other ovarian and sarcoma subtypes, as well as retrospective data. Such studies have shown activity with platinum and taxane combinations [2–6]. Similarly, ifosfamide-based regimens have demonstrated clinical benefit in retrospective studies [7–9], with limited prospective phase II data [10]. Although the optimal chemotherapy regimen for OCS has not yet been defined, an ongoing front-line phase III study for uterine and ovarian carcinosarcomas (GOG-0261, NCT00954174) aims to determine which doublet combination, carboplatin/paclitaxel or ifosfamide/paclitaxel, is superior. However, it is likely that the response rates and survival benefit will continue to be inferior to that of epithelial OC, as it has been historically [6]. As such, novel therapies are needed in order to achieve better outcomes in OCS.

Targeting pathways like vascular endothelial growth factor (VEGF) and the epidermal growth factor (EGF) family (HER) may be beneficial in OCS [11, 12]. Given the rarity of OCS, there are limited data regarding the relevance of these pathways but when uterine carcinosarcoma tumors were analyzed for expression of potentially targetable genes, expression of EGFR and HER-2 was detected in 58.4% and 24.8% of samples (n = 101), respectively, while VEGF expression was found in 100% (n = 30) of patients [11, 13, 14]. In addition, VEGF targeting has demonstrated clinical benefit in large prospective randomized trials (GOG-0218 and ICON-7) of epithelial OC, but no such data exists for OCS [15, 16].

Less is known about the therapeutic role of insulin-like growth factor receptor-I (IGF-1R) inhibition in carcinosarcomas. However, in epithelial OC, the IGF-1R inhibitor BMS-754807 inhibits mesenchymal, epithelial, and hematopoietic tumor growth, and has activity in a xenograft model of epithelial OC [17, 18]. IGF targeting has not been described in OCS but overexpression of IGF-1R and IGF-II was reported in a uterine carcinosarcoma [12]. Thus, there is indirect evidence suggestive of IGF pathway involvement in OCS.

The incidence of OCS makes it difficult to determine the rate of *BRCA1* and *BRCA2* mutations in this disease. However, there is compelling evidence that *BRCA*-wildtype tumors can also demonstrate a BRCA-like phenotype, also referred to as BRCAness [19]. Indeed, measurable responses to olaparib, an inhibitor of poly (ADP-ribose) polymerase (PARP), were achieved in 24% of epithelial OC patients without known BRCA mutations [20]. The etiology of BRCAness is thought to involve the loss of other key genes required for homologous recombination (HR). Using massively parallel genomic sequencing, 4 of 12 OCS tumors demonstrated loss of function mutations in HR genes [21] and it is possible that PARP inhibition may be effective in this disease.

A barrier to achieving better clinical outcomes is the paucity of relevant animal models that recapitulate a patient’s response to therapy. In an effort to overcome this limitation, a biobank of common and rare OC patient-derived xenograft (PDX) models has been developed [22]. In this study, we present the differential response of an OCS to chemotherapy and explore the efficacy of combined targeted therapy.

## Materials and Methods

### Development of Carcinosarcoma Tumorgraft

Mayo Clinic Institutional Review Board (IRB) approved the collection of tissue from surgeries and approved the patient written consent form. Signed consent was scanned into the patient electronic medical record. Surgical tissue from a patient with ovarian carcinosarcoma was immediately taken to the research laboratory and issued a unique identifier in accordance with the Health Insurance Portability and Accountability Act. Tissues were processed as previously described for intraperitoneal establishment and viable storage of human OC tissue [22]. For each study, viably frozen tumorgraft tissue of patient heterotransplant #003 (PH003) was quickly thawed and washed several times in McCoy’s media. Minced OCS (0.1cm^3^ per mouse) was injected intraperitoneally.

All animal studies with female severe combined immunodeficient (SCID) mice (C.B.-17/IcrHsd-Prkdcscid Lystbg; Harlan Laboratories) were conducted with approval from, and in accordance with, the Mayo Clinic Institutional Animal Care and Use Committee, accredited by the American Association of Laboratory Animal Care, which meet or exceed the standards set by the U.S. Department of Agriculture Animal Welfare Act, Public Health Service policy on humane care and use of animals, and the NIH guide on laboratory animal welfare. Six- to nine-weeks old mice weighing 16–25 grams were house in the Mayo Clinic Department of Comparative Medicine animal facility with less than 5 mice per pathogen-free cage. Access to standard rodent chow and water was *ad libitum*. Ambient light, temperature, and humidity were constantly controlled and bedding was routinely changed following standard protocols at Mayo Clinic.

### Efficacy of Cytotoxic Chemotherapy *in vivo*

Efficacy of carboplatin/paclitaxel (C/P) and ifosfamide/paclitaxel (I/P) were directly compared. PH003 was injected into SCID mice and subsequently randomized into three groups for intraperitoneal treatment (n = 13 each): control (saline treatment), C/P (64 and 19 mg/kg, respectively), or I/P (116 and 29 mg/kg, respectively). C/P mice were treated with three weekly doses of carboplatin and paclitaxel while the I/P cohort received three weekly doses of ifosfamide and paclitaxel and two additional daily treatments of single-agent ifosfamide following each doublet, as conducted in GOG-0261 (NCT00954174). For all *in vivo* studies, animals were assessed at least five days per week and a health score was derived from appearance, behavior, and body conditioning as described [23]. Scores ≤6 met criteria for moribund and mice were sacrificed by carbon dioxide inhalation.

### Phosphotyrosine Kinase Blot Array

Receptor tyrosine kinase (RTK) phosphorylation was assessed in snap-frozen tumorgraft tissue and cell culture lysates using the Human Proteome Profiler Array (#ARY001, R&D Systems) following the manufacturers standard protocol. Proteins were extracted with 1% NP-40 lysis buffer containing fresh protease and phosphatase inhibitors (Sigma-Aldrich, St. Louis, MO Cat# P8340 and #P5726) with disruption (tumorgraft tissue only) in a VirSonic Ultrasonic Cell Disrupter 100 (The VirTis Company, Gardiner NY). Protein concentration was determined by BCA Reagents (Thermo Scientific, Waltham, MA) and 50 micrograms of protein was applied to blot arrays overnight at 4°C with agitation. The anti-Phospho-Tyrosine-HRP Detection Antibody was applied for 2hrs at room temperature with agitation and expression was visualized with Chemi Reagent Mix, provided in the kit. Blots were simultaneous exposure to the same x-ray film. Signals were analyzed using ImageJ 1.47v to quantify dot intensity peaks using the rolling-ball method for background subtraction [24]. Blots lacked a housekeeping gene for normalization so only intra-blot relative phospho-protein expression was compared.

### Array Comparative Genomic Hybridization

Array comparative genomic hybridization (aCGH) was performed using the Agilent Human Genome CGH microarray kit 244A with matched-patient reference germline DNA as previously described [22]. Tumorgraft DNA was extracted following the manufacturers protocol for Qiagen AllPrep DNA/RNA mini Kit (#80204) and quantitated on a Thermo Scientific NanoDrop 2000c UV-Vis Spectrophotometer. Test (tumor) and reference (matched patient germline) DNA was labeled with Cy5 and Cy3, respectively, by random priming PCR, hybridized over 24 hrs at 65°C, and analyzed with Agilent Technologies Genomic Workbench 6.5 Lite Edition software. Gains and losses were defined as >4 regional probes with an absolute average log ratio of ≥0.26 for the region.

### Quantitative Polymerase Chain Reaction (QPCR)

To validate copy number aberrations detected by aCGH, tumor and matched patient germline DNA were analyzed by QPCR. *EGFR* primers (Set 1, FW 5’-ccttggcacctttctactgc-3’ and REV 5’-tcaggaagccagctctttgt-3’; Set 2 FW 5′-GGGCAAAGAAGAAACGGAG-3′ and REV 5′-GTCCATCAGTGGGGAGTAAG-3′) were used to amplify 10 ng of genomic DNA in a LightCycler 480 II (Roche Life Science, Indianapolis, IN) with SYBR green as the detection method. Rather than use a single locus as the reference for calculation of ΔΔCT, a multi-copy locus primer kit (Type-it CNV SYBR Green PCR, Cat# 206672) from Qiagen was used to minimize the impact of genome-wide gains and losses expected in tumor DNA. The ratio (R) of the copy number change of *EGFR* in tumor DNA compared to matched germline DNA was calculated by R = 2^(-ΔΔCT)^, where ΔΔCT = ΔCT (Tumor DNA)– ΔCT (Germline DNA) = (CT (EGFR, Tumor DNA)–CT (Reference, Tumor DNA))–(CT (EGFR, Germline DNA)–CT (Reference, Germline DNA)). An R>1 indicates a higher copy number of *EGFR* in the tumor DNA relative to germline.

### Pathway-directed Therapeutics

PH003 OCS was heterotransplanted into SCID mice (n = 13 each cohort) and treated with C/P alone or in combination with either EVRI (pan-HER and VEGF inhibitor, BMS 690514), IGF-1Ri (IGF-1R inhibitor, BMS 754807) or PARPi (poly ADP-ribose polymerase inhibitor, ABT-888). All targeted therapies were purchased from Selleck Chemicals, Houston, TX. EVRI (50 mg/kg) and IGF-1Ri (50mg/kg) was diluted and administered by daily oral gavage as previously described [25, 26] and ABT-888 (25 mg/kg in PEG400:water, 80:20) was administered by daily oral gavage on days 1–3, 8–10, and 15–17. The dose of ABT-888 was chosen based on previous *in vivo* data showing potentiation of chemotherapy at 25 mg/kg/day [27]. However, ensure animals could tolerate a longer treatment course, the schedule was modified to intermittent dosing, which is a dosing schedule used in an ongoing clinical trial in ovarian cancer at Mayo Clinic (NCT01012817).

### Western Blots

Protein extraction and blotting was performed as previously described [28]. Tumor tissue was homogenized in buffer containing 50 mM Tris (pH 7.4), 1 mM EDTA, 150 mM NaCl and proteinase inhibitors (1 μg/ml phenylmethylsulfonyl fluoride, 10 μg/ml aprotinin and 1μg/ml leupeptin). The homogenates were centrifuged at 2,000g for 15 min and 10,000g for 5 min prior to determining concentration using BCA Reagents. Proteins were separated by 10% SDS-PAGE electrophoresis and transferred to Immuno-Blot polyvinylidene difluoride (PVDF) membranes (Bio-Rad, Cat#: 162–0177,). The membranes were blocked with 5% milk in TBS (10 mM Tris-HCl pH 8.0 and 150 mM NaCl) plus 0.05% Tween-20 for 1 hour and then incubated with primary antibodies in 5% milk overnight at 4°C. After washing, blots were incubated with HRP-conjugated secondary antibodies in 5% milk for 1 hour at room temperature. The bands were detected using an ECL kit (Amersham, Arlington Heights, IL) and visualized on x-ray film.

All antibodies were commercially available. Monoclonal anti-PAR polymer antibody (#4335-MC-100-AC, Trevigen, Gaithersburg, MD) was used at 1:1000. Rabbit polyclonal anti-total Akt antibody (#9272, Cell Signaling, Danvers, MA) was diluted 1:1000 and anti-phospho-Akt antibody (#9271, Cell Signaling) was diluted 1:2000. Mouse monoclonal anti-actin antibody (#A3853, Sigma-Aldrich, St. Louis, MO) was used at 1:1000. Anti-mouse (#074–1806) or anti-rabbit (#074-15-061) secondary antibodies were used at 1:2000 (KPL Inc., Gaithersburg, MD).

### Next Generation Sequencing and Bioinformatics

Genomic DNA from fresh untreated PDX tissue was extracted as described above for aCGH. Samples were enriched using the Ion AmpliSeq Cancer Hotspot Panel (Life Technologies, Grand Island, NY) and prepped using the TruSeq Nano Library prep (Illumina, San Diego, CA) before sequencing as Paired End 150 reads on the Illumina MiSeq. To account for mouse genomic DNA, the Xenome tool [29] was used to classify sequence read data as either human or mouse genome. Only the human paired-end Illumina reads were analyzed using Genome_GPS, an analysis pipeline for DNA sequencing data at Mayo Clinic. Raw FASTQ formatted reads were aligned using bwa-mem version 0.7.10 [30] against reference genome (hg19), and the aligned Sequence Alignment Map (SAM) files were converted to a coordinate sorted binary SAM (BAM) file using SAMtools 0.1.19 [31]. The BAM files were then processed through Genome Analysis Toolkit 2.7 (GATK, Broad Institute, Cambridge, MA, USA). The raw variants were called using GATK's UnifiedGenotyper walker version 2.7 with default parameters. Significant variants were called if the effect was non-synonymous and contained ≥249 high quality reads.

### Statistical Analysis

The sample sizes in each *in vivo* study had 89% power to detect a 20% difference in mean tumor mass between control and treated animals. Survival curve differences were analyzed by log-rank test with significance defined by p < 0.05. To analyze group data, differences were determined using the Student’s t test (two tailed) or ANOVA with P ≤ 0.05 considered statistically significant. Pearson’s correlation was used to assess association between continuous variables.

## Results

### Defining the Maximum Tolerated Dose of Chemotherapy

Although dose and schedule for targeted therapies were chosen based on published literature, the maximum tolerated dose of intraperitoneal carboplatin/paclitaxel (C/P) or ifosfamide/paclitaxel (I/P) was not well defined. Mice bearing palpable tumors were divided into two arms (n = 4 each) and treated with intraperitoneal C/P or I/P at starting doses reported in the literature for mono-therapy and adjusted to account for their simultaneous administration [32–36] (Table 1). The maximum tolerated dose was determined using a standard ‘3 + 3’ design commonly utilized in phase 1 clinical trials [37]. Dose-limiting toxicity (DLT) was defined as a treatment dose that resulted in a health score of ≤6 or weight loss greater than 20% of baseline. For the C/P group, one mouse developed a DLT at level two and after expansion to a total of seven mice, additional DTL was observed and only dose level 1 was deemed tolerable at 64 mg/kg carboplatin and 19 mg/kg paclitaxel. For the I/P group, no DTLs were observed in >1 mouse at any dose level so 116 mg/kg ifosfamide and 29 mg/kg paclitaxel (level 3) was deemed tolerable.

**Table 1:** Dosing algorithms for dose finding portion of study.

Carboplatin/Paclitaxel Dosing Algorithm		
Dose Level	Carboplatin mg/kg	Paclitaxel mg/kg
0	51	15
1*	64	19
2	80	24
3	96	29
**Ifosfamide/Paclitaxel Dosing Algorithm**		
Dose Level	Ifosfamide mg/kg	Paclitaxel mg/kg
-1	64 Day 1	15
0	80 Day 1	19
1*	80 Day 1,2,3	19
2	96 Day 1,2,3	24
3	116 Day 1,2,3	29

*Starting dose level

### Histologic Evaluation of Tumor Tissue

**Fig 1:**
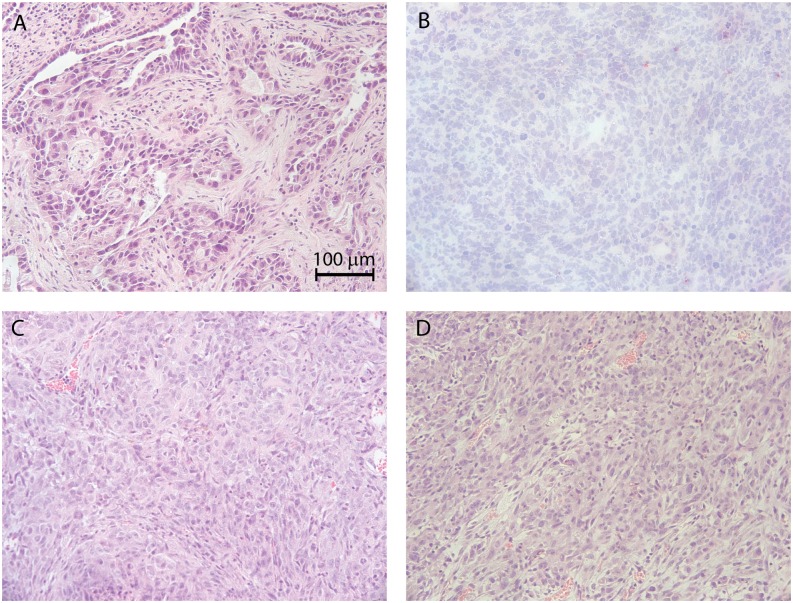
Hematoxylin and Eosin staining of PH003. Patient (A & B) and tumorgraft (C & D) tissues are shown. Images were captured with a 40x objective.

To determine the histologic similarity between the PH003 patient and PDX tumor tissue, hematoxylin and eosin staining was performed on formalin-fixed, paraffin embedded 5–6 μm sections. Two different source tumor regions were examined and exhibited variability in contribution from mesenchymal and carcinoma components (Fig 1A and 1B). Four different PH003 xenografts were examined and representative images (Fig 1C and 1D) show a closer resemblance to the confluent carcinoma pattern of the patient (Fig 1B).

### Semi-Quantitative Assessment of Tumor Size *in vivo*

**Fig 2:**
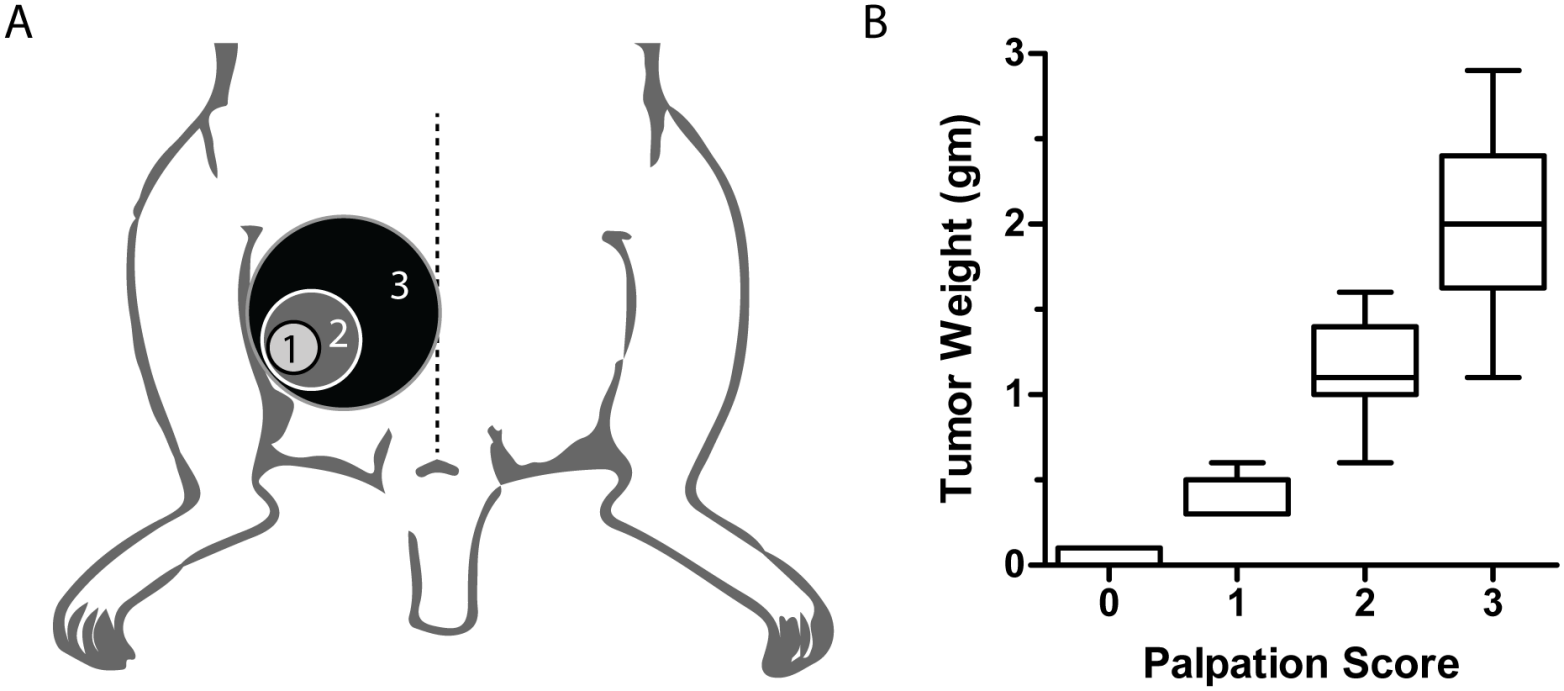
Correlation of abdominal palpation score and tumor weight at necropsy. (A) The palpation score was based on tumor size relative to the mouse hemi-pelvis. (B) Palpation score prior to necropsy correlated with tumor weight at necropsy (Pearson r = 0.8965, two-tailed p <0.0001).

OCS model PH003 tends to form a discrete mass(s) rather than carcinomatosis, making assessment of intra-abdominal tumor burden possible by direct palpation. Since serial *in vivo* assessment of intraperitoneal tumor size is difficult by caliper measurements, a semi-quantitative study was conducted to determine the utility of an abdominal-pelvic tumor palpation scoring system, based on the palpable tumor size relative to the mouse hemi-pelvis: zero = non-palpable, 1 is < 25%, 2 is 26–49%, and 3 is >50% (Fig 2A). When tumor diameter was scored prior to necropsy and compared with post-necropsy tumor weight, strong correlation was observed (Pearson r = 0.8965, two-tailed p <0.0001 (Fig 2B).

### Response to Cytotoxic Chemotherapy *in vivo*

**Fig 3:**
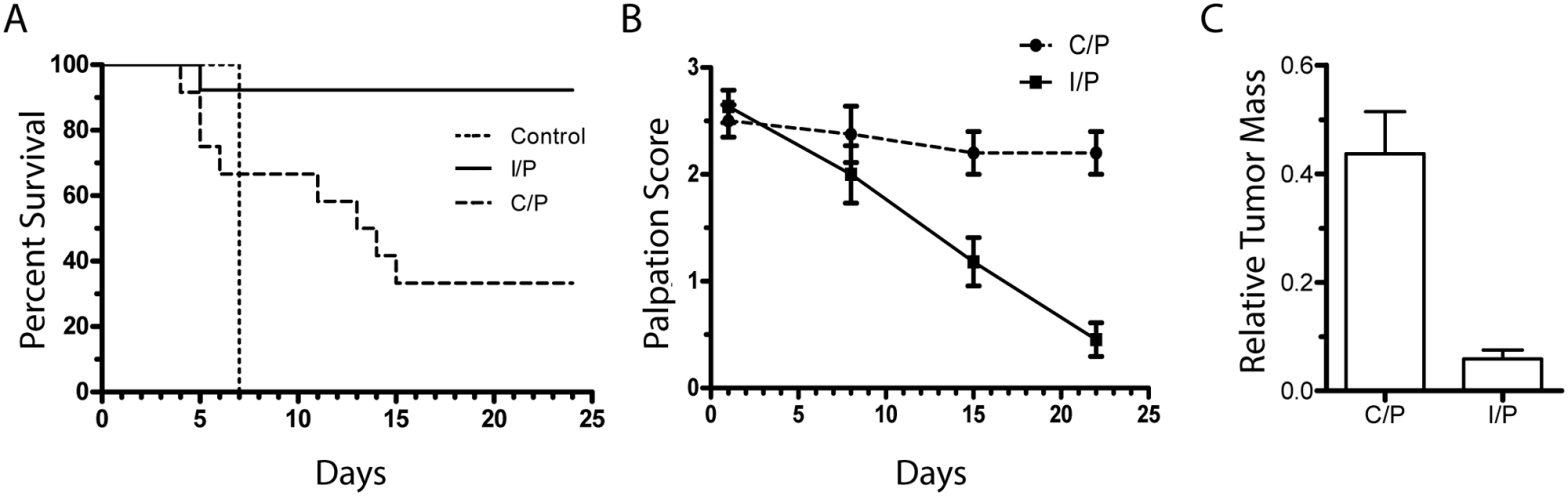
Tumorgraft response to treatment in vivo. Mice were randomized to receive (n = 13 each group) either saline, carboplatin and paclitaxel (C/P), or ifosfamide and paclitaxel (I/P). (A) Kaplan-Meier Curve demonstrates survival. The C/P and I/P curves were significantly different, log-rank test p = 0.0027. (B) Tumor palpation score over time reflects the change in tumor size with treatment. (C) Tumor weights in each cohort were normalized against the final mean control weight obtained at necropsy on day 7 (which represents the maximum-allowed tumor burden). The difference between C/P and I/P relative tumor mass was significant (two-tailed t test, p <0.0001).

When engraftment was evident by palpation, mice were randomized to receive saline control, C/P, or I/P at doses defined above. The primary endpoint was tumor mass at necropsy but overall survival and tumor palpation score were recorded as secondary endpoints. All mice had a health score [23] of 11+ on day one and were sacrificed at a score ≤6 or if they had greater than 20% weight loss. Due to rapid growth of OCS PH003, control mice were moribund by treatment-day 7 (Fig 3A). Animals treated with C/P exhibited improved median survival to 13.5 days while the I/P cohort had only one moribund animal during the study period and the median survival was not reached. The difference between treatment arms was significant (log-rank test, p = 0.0027). The serial decline in palpation scores observed with I/P treatment was consistent with improved survival, indicating tumor regression over time (Fig 3B). The stable palpation score seen in the C/P-treated group was also consistent with survival data as moribund mice with larger tumors were sacrificed over time. When normalized to the mean control tumor mass measured at necropsy (day 7), the difference in final tumor mass at necropsy between C/P (0.44 +/-0.078) and I/P (0.059 +/- 0.016) cohorts was significant (p < 0.0001, two-tailed t test) (Fig 3C).

### Activation of Oncogenic Pathways

**Fig 4:**
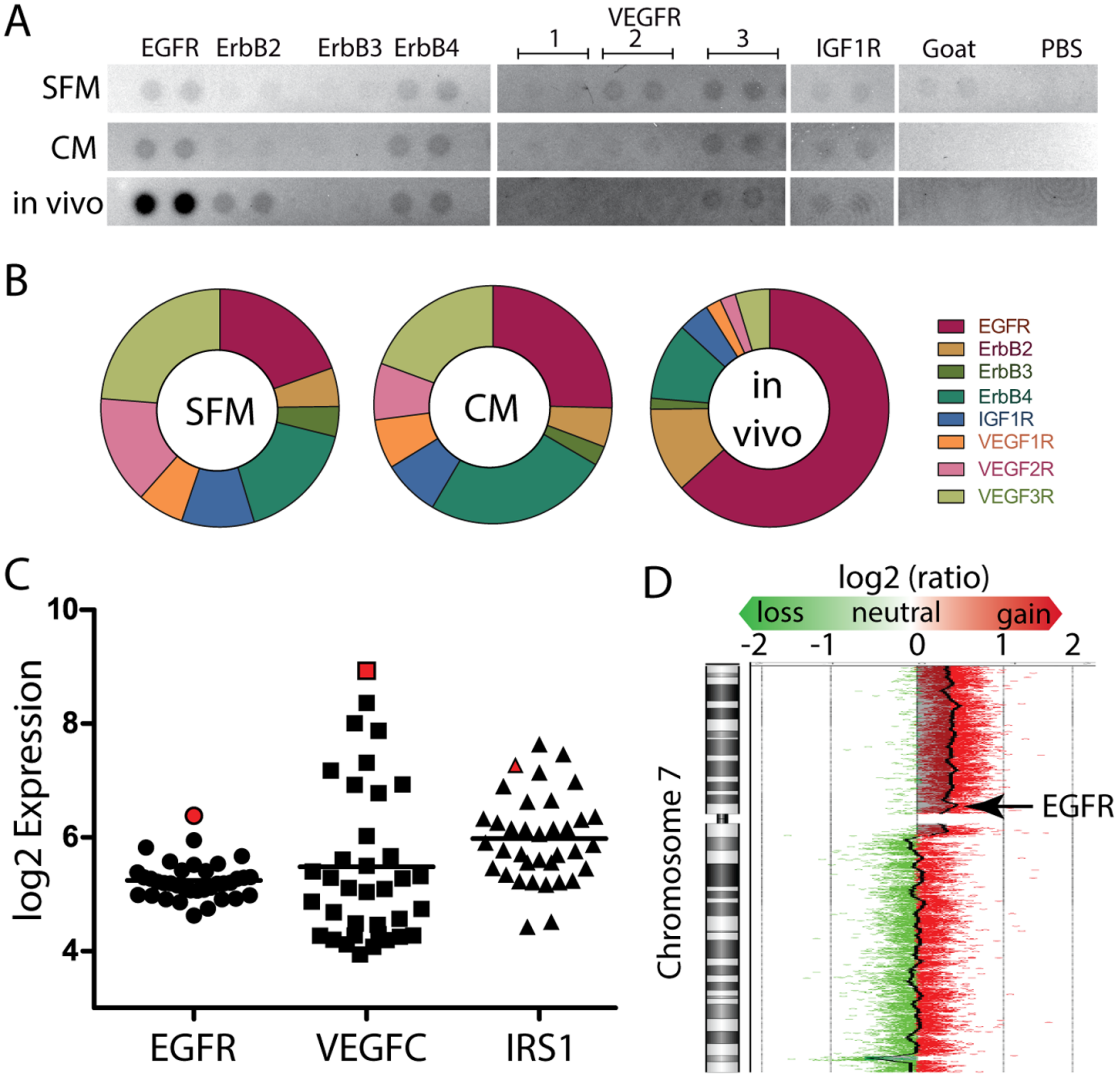
Potential targets of novel therapies. (A) Receptor tyrosine kinase (RTK) dot blot array. Goat IgG and phosphate buffered saline (PBS) are background controls. Each RTK is blotted in duplicate. (B) The proportion of signal contributed by select RTKs in serum free media (SFM), complete media (CM), and in tissues harvested after engraftment in a mouse (*in vivo*). (C) Microarray analysis for expression of EGFR, VEGFC, and IRS1 in model PH003 (red shape) and 35 other ovarian patient-derived tumorgraft models (black shapes). (D) Array comparative genomic hybridization showing chromosome 7. Red dots represent probes from the tumor genome and Green dots represent probes from the patient’s germline DNA. A balance of Red and Green probes signifies equal representation of tumor and germline DNA in the q arm. The p arm shows an unbalanced representation of tumor genomic DNA (log2 ratio 0.399 compared to germline). The bold vertical jagged line shows the average log2 ratio of tumor and germline DNA along chromosome 7.

Since platinum-based doublet chemotherapy is a standard of care for OCS and the observed *in vivo* response to C/P was inferior to I/P, exploratory analysis of oncogenic pathways was performed in order to find potentially targetable pathways with an ultimate goal of augmenting treatment efficacy with C/P. An RTK array was performed to assess for phosphorylated RTKs in actively proliferating untreated control PDX tissue. Representative images are shown for pathways relevant to OCS: the epidermal growth factor family, vascular endothelial growth factor receptor, and insulin-like growth factor receptor 1 (Fig 4A). PDX tissue showed a disproportionately higher level of phosphorylated HER family of receptors, EGFR in particular, suggesting a potential role for HER inhibition *in vivo*. In the absence of a relevant control tissue, PH003 PDX tissue was minced and passed through a cell strainer for short-term culture and the RTK phosphorylation profile was assessed during serum-starvation (serum-free McCoy’s media for 24 hours prior to harvest) and after overnight starvation followed by 15 minutes of complete media prior to harvest. The proportion of phosphorylated RTKs remained largely unchanged between serum-starved and serum-fed states (Fig 4A and 4B), suggesting a potential role for EGFR activation *in vivo* but not *in vitro*. To compile additional evidence to support HER targeting, Affymetrix microarray data was queried from 36 tumorgraft models, including PH003, which was previously reported [38] and publically available (Gene Expression Omnibus # GSE66667). PH003 PDX was found to have the highest relative expression of EGFR (Fig 4C). In addition, aCGH showed amplification of chromosome 7p, containing *EGFR* with a log2 ratio of 0.399, or a 1.32-fold change in tumor DNA relative to germline DNA (Fig 4D). This amplification was validated by QPCR using two separate *EGFR* primers showing a mean 1.26-fold (+/- 0.042 SE) difference in *EGFR* copy number relative to the source patient germline DNA. Inhibition of vascular endothelial growth factor in ovarian cancer is clinically relevant and the high relative expression of VEGFC implicates this pathway in PH003 growth (Fig 4C). Since IRS-1 is an adaptor protein that links IGF-1R to downstream signal transduction, its high expression relative to other tumorgraft models implicates the IGF system as a potential target (Fig 4C).

Given the preliminary evidence for activity of PARP inhibitors in both *BRCA* mutant and wild-type ovarian cancer, we evaluated PARP inhibition [39, 40]. However, given the lack of responsiveness of the source patient to platinum—based chemotherapy and lack of markers by aCGH and DNA microarray to suggest deficiencies in homologous recombination, our hypothesis was that PH003 would lack responsiveness.

### Efficacy of Concomitant Cytotoxic and Targeted Therapy

**Fig 5:**
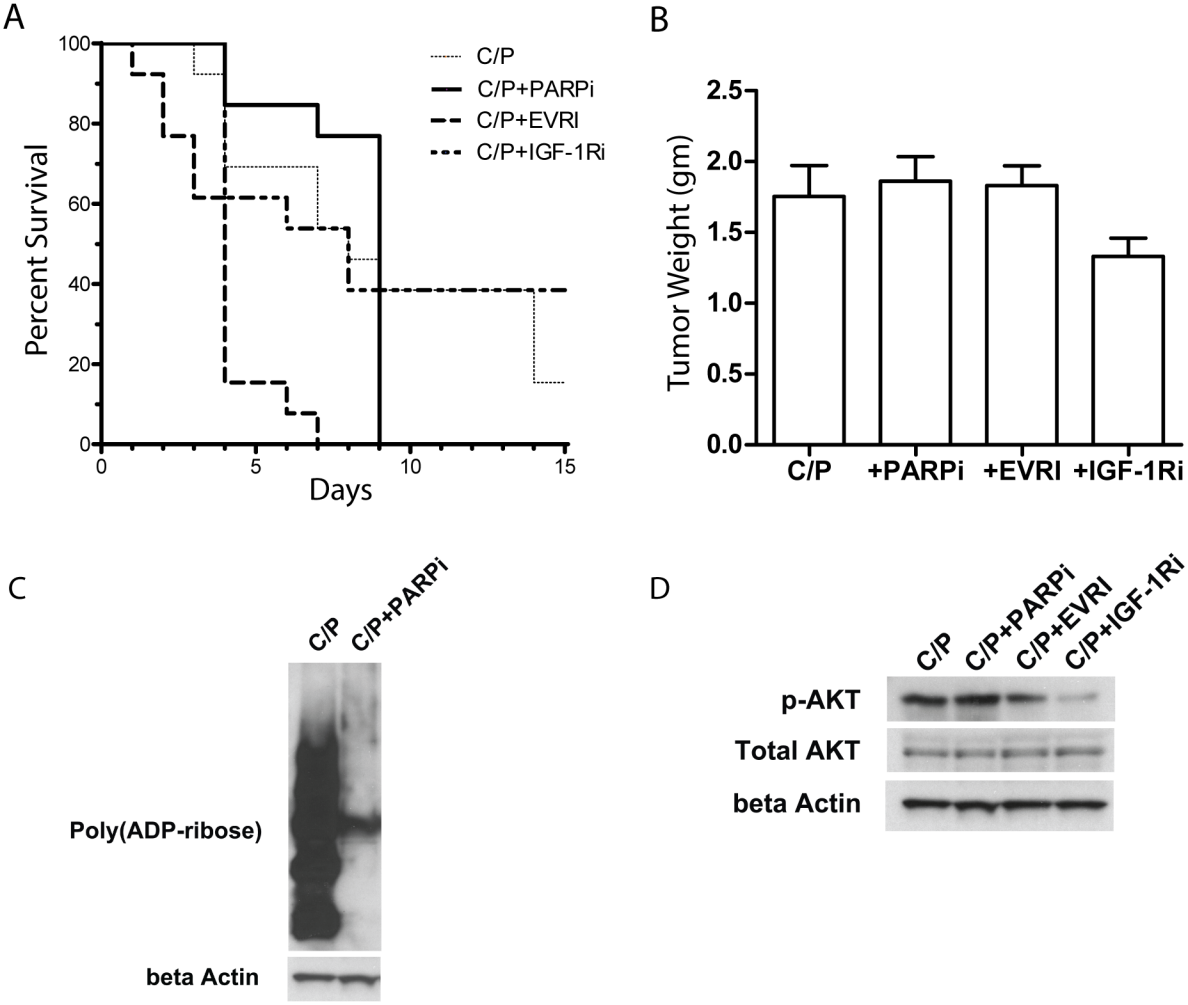
Tumorgraft response to carboplatin and paclitaxel with or without targeted therapy. (A) Kaplan-Meier Curve showing animal survival (log-rank test p = 0.8456). (B) There was no difference in final tumor weight between groups (one-way ANOVA p = >0.05 each comparison), which was the primary endpoint. (C) Western blot of PAR in tumor treated with ABT-888. (D) Western blot of total and phospho-AKT in tumors after treatment. Abbreviations: Carboplatin and paclitaxel (C/P), EVRI (pan-HER and VEGF inhibitor BMS 690514), IGF-1Ri (BMS 754807), PARPi (ABT-888).

Since the *in vivo* response to C/P was significantly inferior to I/P, the concomitant use of targeted therapy was tested. Mice were heterotransplanted with OCS tumorgraft tissue as above and after palpable tumors were present, randomization occurred into one of four cohorts, n = 13 each: C/P alone, C/P + EVRI, C/P + IGF-1Ri, or C/P + PARPi. The rationale for using EVRI (pan-HER and VEGF inhibitor) was based on previous work showing improved efficacy with dual-pathway targeting in a xenograft model of epithelial OC [25]. Primary and secondary endpoints were tumor mass at necropsy and overall survival, respectively. No combination therapy significantly extended survival relative to C/P alone (Fig 5A). In addition, there were no differences in final tumor weight relative to C/P at necropsy (one-way ANOVA with Dunnett’s Multiple Comparisons, p >0.05) (Fig 5B).

To explore the potential reasons for this lack of response, post-treatment tumor tissue was analyzed by Western blot for downstream markers of on-target effects. With regard to PARP inhibition, ABT-888 effectively inhibited PAR polymer formation in PH003 PDX tissue (Fig 5C). Treatment with EVRI or IGR-1Ri resulted in downregulation of p-AKT relative to chemotherapy-only controls (Fig 5D). Since mutations may confer resistance to targeted therapies, next-generation sequencing of select genes was performed. PH003 exhibited a non-synonymous *KRAS* mutation at c.35G>A (p.Gly12Asp based on RefSeq NM_004985, rs121913529). In addition, a pathogenic mutation in *PIK3CA* (c.1258T>C) was discovered (p.Cys420Arg based on RefSeq NM_006218, rs121913272). Although a stop codon was detected in *PTEN*, the tumor was homozygous for this mutation and the biological significance is uncertain (S1 Table). A mutation in *CTNNB1* was also detected but the read depth fell below our threshold for significance. A *TP53* mutation was also discovered at c.347G>A (p.Arg116Gln based on RefSeq NM_001126115, rs11540652).

## Discussion

Ovarian carcinosarcoma is one of the least common but most aggressive forms of OC with no prospective, randomized data to define a standard chemotherapy treatment. This is largely due to the rarity of OCS, which imposes significant challenges with regard to patient accrual to clinical trials [36]. To address such challenges, an ongoing project to collect and characterize rare and common OC tumor types is underway [22]. The first *in vivo* efficacy studies of an OCS tumorgraft is reported herein. We show that I/P was superior to C/P in this OCS model and there was limited efficacy of rationally-selected targeted therapies.

With a single OCS model, the current study was not designed to show superiority of C/P or I/P in ovarian carcinosarcomas. However, it has been shown that *in vivo* patient-derived xenograft responses to therapy correlate to the matched patient experience [22, 41, 42]; such models provide an opportunity to compare the efficacy of different chemotherapy combinations in individual PDX models, and by extension, in individual patient. Although its unknown if I/P would have been more effective in the patient from which PH003 was derived, the patient was treated with C/P and developed bone metastases within two months of the last carboplatin/paclitaxel dose, indicative of chemotherapy resistance and consistent with the PH003 PDX response.

Since the overall prognosis for OCS is inferior to other ovarian cancers treated with standard chemotherapy, novel targeted agents are needed. In this study, a rationalized approach to drug selection was attempted. However, the lack of response to inhibitors of EGFR, VEGFR, IGF-1R, and PARP indicates that gene expression and amplification profiling alone are not sufficient to predict response. Indeed, further molecular characterization showed that PH003 harbors a *KRAS* mutation in codon 12, p.Gly12Asp, which was previously described in lung [43] and colon cancers [44], and is associated with decreased efficacy of EGFR inhibitors afatinib and gefitinib [45]. As such, p.Gly12Asp may explain the lack of response from EVRI. Mutations reported herein for *KRAS*, *PIK3CA*, and *TP53* are consistent with previous studies on patient samples [46] and supports the clinical relevance of this OCS model.

Assessment of intraperitoneal *in vivo* tumor response during treatment is central to PDX-based studies. Although techniques such as ultrasound [22] and bioluminescence [25] are well established, such methods require specialized training and/or equipment. Alternatively, the current study supports the use of a physical-exam based scoring method to supplement final tumor weight and animal survival as endpoints.

The histologic characteristics of common ovarian patient-derived xenograft subtypes (e.g. serous and endometrioid) have been described previously [22, 42, 47–52] but there is a paucity of PDX models for OCS. The PH003 PDX shows a predominately carcinomatous pattern with less contribution from mesenchymal components when compared to the source tumor. Similar histologic observations were also seen in another OCS PDX model, JoN xenografts [53]. However, JoN xenografts were developed with an interim *in vitro* culturing step prior to heterotransplantation in mice and it is possible that the sarcomatous components were selectively excluded. Since PH003 was directly heterotransplanted and the stromal components of xenograft tissue are derived from the murine host [22], the rapid growth of PH003 may not permit concurrent outgrowth of the mesenchymal component.

As interest continues to grow for the utilization of patient-derived tumor xenografts (tumorgrafts) in drug development [54], there is hope for the identification of novel therapies in rare cancers such as OCS. Model PH003, and future OCS tumorgraft models, may be helpful in this regard as the number of rare ovarian tumorgrafts continue to grow in the Mayo Clinic ovarian cancer PDX bank [22].

## Supporting Information

S1 Table Next-generation DNA sequencing results of tumorgraft tissue, sorted by read depth. CHROM: The chromosome; POS: The reference position, with the 1st base having position 1; DP: Approximate read depth; some reads may have been filtered; QUAL: Phred-scaled quality score for the assertion made in ALT using formula -10log_10*P where P = base-calling error probability. REF: The reference base; ALT: alternate non-reference allele; AF: allele frequency in the sample; NSC: non-synonymous coding. * Stop codon gained.
(DOCX)
