# Supplementary material for: Conventional Chemotherapy and Oncogenic Pathway Targeting in Ovarian Carcinosarcoma Using a Patient-Derived Tumorgraft
Source: PLoS One. 2015 May 11;10(5):e0126867. doi: 10.1371/journal.pone.0126867 (PMC4427104; doi:10.1371/journal.pone.0126867)
Supplement: S1 Table — CHROM: The chromosome; POS: The reference position, with the 1st base having position 1; DP: Approximate read depth; some reads may have been filtered; QUAL: Phred-scaled quality score for the assertion made in ALT using formula -10log_10*P where P = base-calling error probability. REF: The reference base; ALT: alternate non-reference allele; AF: allele frequency in the sample; NSC: non-synonymous coding. * Stop codon gained. (DOCX) [file pone.0126867.s001.docx]

Supporting Information

| **Gene Name** | **CHROM** | **Base POS** | **REF** | **ALT** | **ALT AF** | **DP** | **QUAL** | **ALT Amino Acid Change** | **ALT Functional Class** | **ALT Effect** |
| --- | --- | --- | --- | --- | --- | --- | --- | --- | --- | --- |
| KRAS | 12 | 25398284 | C | T | 0.5 | 250 | 24685.47 | G12D | MISSENSE | NSC |
| TP53 | 17 | 7577538 | C | T | 1 | 250 | 26318.39 | R116Q | MISSENSE | NSC |
| PIK3CA | 3 | 178927980 | T | C | 1 | 249 | 8758.63 | C420R | MISSENSE | NSC |
| KRAS | 12 | 25378673 | C | G | 0.5 | 62 | 22557.62 | V109L | MISSENSE | NSC |
| KRAS | 12 | 25380239 | C | G | 0.5 | 55 | 923.43 | R73S | MISSENSE | NSC |
| PTEN | 10 | 89717642 | A | T | 0.5 | 11 | 418.33 | K223* | NONSENSE | STOP GAINED |
| CTNNB1 | 3 | 41266163 | G | A | 0.5 | 3 | 128.11 | E54K | MISSENSE | NSC |

S1 Table. Next-generation DNA sequencing results of tumorgraft tissue, sorted by read depth.

CHROM: The chromosome

POS: The reference position, with the 1st base having position 1

DP: Approximate read depth; some reads may have been filtered

QUAL: Phred-scaled quality score for the assertion made in ALT using formula -10log_10*P where P= base-calling error probability.

REF: The reference base

ALT: alternate non-reference allele

AF: allele frequency in the sample

NSC: non-synonymous coding

* Stop codon gained
